# A survey of mathematical models of human performance using power and energy

**DOI:** 10.1186/s40798-019-0230-z

**Published:** 2019-12-27

**Authors:** Vijay Sarthy M. Sreedhara, Gregory M. Mocko, Randolph E. Hutchison

**Affiliations:** 1 Department of Mechanical Engineering, Clemson University, 243 Fluor Daniel EIB, Clemson, SC 29634-0921 USA; 2 Department of Health Sciences, Furman University, Greenville, SC 29613 USA

**Keywords:** Human performance modeling, Fatigue, Time to exhaustion, Energy expenditure, Recovery, Critical power

## Abstract

The ability to predict the systematic decrease of power during physical exertion gives valuable insights into health, performance, and injury. This review surveys the research of power-based models of fatigue and recovery within the area of human performance. Upon a thorough review of available literature, it is observed that the two-parameter critical power model is most popular due to its simplicity. This two-parameter model is a hyperbolic relationship between power and time with critical power as the power-asymptote and the curvature constant denoted by *W*′. Critical power (CP) is a theoretical power output that can be sustained indefinitely by an individual, and the curvature constant (*W*′) represents the amount of work that can be done above CP. Different methods and models have been validated to determine CP and *W*′, most of which are algebraic manipulations of the two-parameter model. The models yield different CP and *W*′ estimates for the same data depending on the regression fit and rounding off approximations. These estimates, at the subject level, have an inherent day-to-day variability called intra-individual variability (IIV) associated with them, which is not captured by any of the existing methods. This calls for a need for new methods to arrive at the IIV associated with CP and *W*′. Furthermore, existing models focus on the expenditure of *W*′ for efforts above CP and do not model its recovery in the sub-CP domain. Thus, there is a need for methods and models that account for (i) the IIV to measure the effectiveness of individual training prescriptions and (ii) the recovery of *W*′ to aid human performance optimization.

## Key Points

- Mathematical models of human energy expenditure and recovery present opportunities in quantifying, evaluating, and optimizing performance.
- Established models are focused on energy expenditure and the available models that focus on recovery need refinement to be used in real-time performance optimization.
- Existing models derived from group data neglect the intra-individual variability (IIV) which is critical in evaluating improvements and optimizing performance at the individual level.

## Background

The study of human fatigue and energy expenditure, and to a lesser degree recovery, has been a focal area of research since the early 1900s. Seminal works in the fields of exercise physiology and performance modeling by A. V. Hill [1], Monod and Scherrer [2], and Ward-Smith [3] have laid the groundwork for modeling energy expenditure during prolonged exertion. Recently, researchers have developed formal mathematical models that aid in better management of performance and push limits of human endurance. Most available models have originated from cycle ergometer tests [4] due to the ease of measuring power in cycling and then applied to other forms of exercise like running [5], swimming [6], and rowing [7]. Additionally, most of these models focus on energy exertion with only a few publications that focus on energy recovery, which could give us valuable insight into the physiological underpinnings of fatigue, recovery, and ultimately optimizing performance. Furthermore, developing models of human performance and fatigue lead to applications such as mission planning of soldiers and investigating the influence of physical activity on cardiovascular and overall health of a human being.

The purpose of this review is to survey the available literature and summarize all the existing power-based models of human performance. Additionally, this review will identify potential research opportunities to advance the field of human performance in terms of modeling individual variance seen in performance metrics, expenditure and recovery models of work capacity, the potential use of performance models in team sports, the influence of exercise on health, and the use of wearable sensors in mitigating the dependency on laboratory equipment.

## Main Text

### Modeling Performance Using Power

There are several definitions of fatigue across researchers that limit the ability to measure and develop mathematical models [8, 9]. For the purpose of this manuscript, fatigue is defined as an exercise induced progressive loss of the ability to sustain maximum power (energy exertion) over a desired duration of time [8–12]. Thus, fatigue is a dynamic process that leads to a drop in the required exercise intensity, which eventually leads to termination of exercise due to exhaustion [13–17]. Exercise intensity is generally categorized as severe, heavy, or moderate [18, 19] based on blood lactate levels [20], maximum oxygen uptake (V̇O_2max_) [21, 22], or power output [22]. Maximal lactate steady state (MLSS) is often used to categorize exercise intensity, which is defined as the highest blood lactate concentration that can be maintained without further accumulation during sub-maximal work [23, 24]. The exercise intensity associated with MLSS indicates the highest intensity that can be supported by aerobic mechanisms [23, 25]. Several methods have been developed to determine MLSS; however, all of them involve taking blood samples and measuring the lactate concentration. Critical power (CP), a theoretical power level which a human can maintain indefinitely [2], is shown to be in close vicinity to the power at which MLSS occurs [26–28]. Moreover, the oxygen uptake (V̇O_2_) and blood lactate levels have been shown to attain a steady state during exercise below CP and hence can be classified as either moderate (below lactate threshold) or heavy (from lactate threshold to CP) intensity [22, 29]. However, exercise above CP is categorized as severe intensity because V̇O_2_ and blood lactate levels cannot attain a steady state [22]. Thus, CP represents the boundary between heavy and severe intensity exercises [30] and provides a convenient and non-invasive way of determining exercise intensity [22, 29]. Furthermore, researchers opine that CP could be the most important fatigue threshold to determine exercise intensity and is the gold-standard to determine the maximal metabolic steady state compared to other parameters such as MLSS, %V̇O_2_, lactate threshold (LT), or gas exchange threshold (GET) as it enables population level standardizations [17, 31].

The critical power concept was introduced by Monod and Scherrer [2] using a linear relationship between total work done and time-to-exhaustion. Monod and Scherrer’s work was based on A. V. Hill’s [1] observations pertaining to athletic records in different sports. Monod and Scherrer coined the terms critical power (CP) and limit work (*W*_Lim_). They defined CP as the power output that an athlete can generate indefinitely and *W*_Lim_ as the total work done until exhaustion at a constant work-rate above CP related by a linear relationship given by:

1 $$ {W}_{\mathrm{Lim}}=a+b\cdot {t}_{\mathrm{Lim}} $$

where “*a*” is an energy reserve in the units of work (Joules) and the constant “*b*” is the critical power in Watts, and *t*_Lim_ is time-to-exhaustion in seconds. Monod and Scherrer derived a hyperbolic form for *t*_Lim_ by substituting *W*_Lim_ as:

2 $$ {W}_{\mathrm{Lim}}=P\cdot {t}_{\mathrm{Lim}} $$

Using Eq. 2 and transforming Eq. 1 as:

3 $$ {t}_{\mathrm{Lim}}=\frac{a}{P-b} $$

where *P* is power in watts. Moritani and colleagues [4] extended the critical power concept to cycling using a series of cycle ergometry tests and called the term “*a*” as anaerobic reserve deriving the linear relationship between *P* and 1/*t*_Lim_ from Eq. 3 given by:

4 $$ P=\frac{a}{t_{Lim}}+b $$

Whipp and colleagues [32] then fit a hyperbolic curve between *P* and *t*_Lim_ with a time asymptote at a power level that is equal to CP and denoted the anaerobic reserve term as *W*′. The anaerobic reserve term *W*′ has since been referred to as anaerobic work capacity (AWC), and these two terms have been used interchangeably. However, it has been shown that *W*′ is not equal to AWC and the two terms should not be used interchangeably [17, 33]. Additionally, it should be noted that *W*′ (pronounced *W* prime) may lead to confusion in mathematical modeling as it is common notation to use “prime” to represent the first derivative with respect to time. Rewriting Eq. 4 by replacing “*a*” with *W*′ and “*b*” with CP yields the following relationship:

5 $$ P= CP+\frac{W\hbox{'}}{t_{\mathrm{Lim}}} $$

**Fig. 1:**
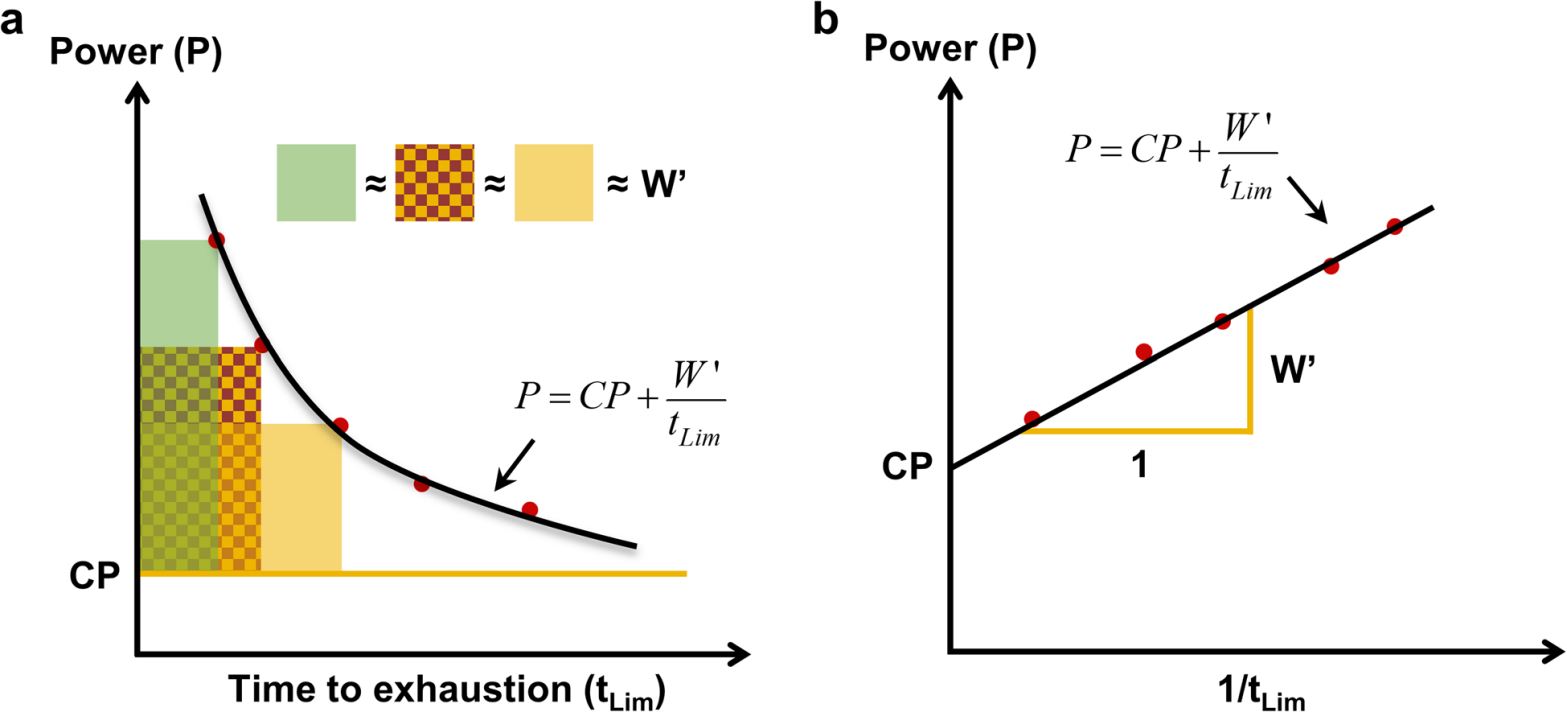
The two-parameter model. **a** The hyperbolic form and **b** the linear transformation with critical power (CP) as the ***y***-intercept and curvature constant (*W*′) as the slope

Equation 5, widely regarded as the two-parameter model, has been transformed to its linear form, first seen in [4] and later in [2, 34–36], by plotting power versus 1/*t*_Lim_ with CP and *W*′ representing the *y*-intercept and slope respectively as shown in Fig. 1. The CP concept has been applied to running [5], swimming [6], and rowing [7] with analogous parameters such as critical velocity (CV) and distance capacity (*D*′) instead of CP and *W*′ respectively.

**Fig. 2:**
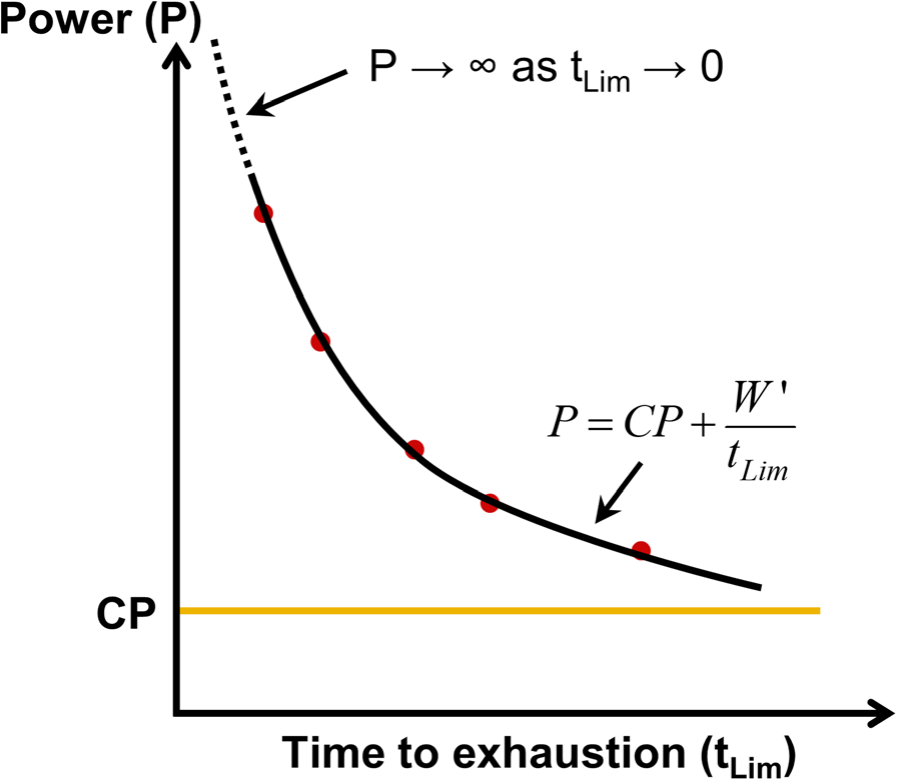
The two-parameter model and its limitations. As *t*_Lim_ tends to 0, *P* tends to ∞, and critical power (CP) is the power asymptote at *t*_Lim_ = ∞

A limitation of the CP concept described by Eq. 5 is that as *t*_Lim_ approaches 0, *P* tends to infinity (see Fig. 2). This is not realistic as there is a limit to the instantaneous maximum power that a human can produce [37, 38]. Moreover, Josephson [39] states that the maximum power output for a muscle occurs at 30% of its maximum shortening velocity (*V*_max_). It takes a short duration of time for the muscle to reach 0.3 *V*_max_ starting from rest. Therefore, it may beneficial to define the instantaneous maximum power as the average power-output for one crank rotation [40]. Additionally, some publications have reported that the average duration for which the CP can be maintained is less than an hour [41–44], while others have reported that it can be maintained for approximately over an hour [45, 46]. D. W. Hill [35] suggests that the end point of the tests proposed to the subjects in these studies, i.e., 24–30 min in [47, 48] and 60–90 min in [41, 45] may have influenced the outcome.

Ward-Smith [3] proposed a model to address these limitations that was able to predict sprint performance between 100 m and 10,000 m. Ward-Smith’s model was derived from the first law of thermodynamics incorporating both the anaerobic and aerobic contributions to the power generated given by:

6 $$ P(t)=\left({P}_{\mathrm{max}}-R\right)\cdot {e}^{\left(-\lambda \cdot t\right)}+R $$

where *P*_max_ is the maximum available power from the anaerobic mechanism, *R* (analogous to CP) is the maximum rate of energy release (power) from the aerobic mechanism, and *λ* represents the decay of power with time *t*. Equation 6 is a simplified version of the model presented by Ward-Smith. The complete version can be found in [3]. Hopkins and colleagues [49] proposed a similar model for treadmill running with inclinations instead of power given by:

7 $$ {I}_t={I}_{\infty }+\left({I}_0-{I}_{\infty}\right)\cdot {e}^{\left(-t/\tau \right)} $$

where *I*_t_ is the inclination at time *t*, *I*_∞_ is the inclination that corresponds to infinite time (similar to CP), *I*_0_ is the instantaneous maximum inclination (synonymous with *P*_max_), and *τ* is a time constant.

Peronnet and Thibault [50] built on Ward-Smith’s work and proposed a model to predict race performances in the range of 60 m to a full marathon. Their main assumption was that the maximum aerobic power (analogous to CP) is sustainable for approximately 7 min as opposed to indefinitely as suggested by Eq. 5. The model is given by:

8 $$ {\displaystyle \begin{array}{l}{P}_T=\frac{S}{T}\left[1-{e}^{\left(-T/{k}_2\right)}\right]+\frac{1}{T}\underset{0}{\overset{T}{\int }}\left[\mathrm{BMR}+B\cdot \left(1-{e}^{\left(-t/{k}_1\right)}\right)\right]\; dt\\ {}(i)\;T<{T}_{\mathrm{MAP}}\Big\{\begin{array}{c}S=A\\ {}B=\mathrm{MAP}-\mathrm{BMR}\end{array}\\ {}(ii)\;T>{T}_{\mathrm{MAP}}\Big\{\begin{array}{c}S=A+A\cdot f\cdot \ln \left(T/{T}_{\mathrm{MAP}}\right)\\ {}B=\left(\mathrm{MAP}-\mathrm{BMR}\right)+E\cdot \ln \left(T/{T}_{\mathrm{MAP}}\right)\end{array}\;\end{array}} $$

where *P*_T_ is the power at any time *T*, *k*_1_ and *k*_2_ are respective time constants to account for the kinetics of aerobic and anaerobic metabolism, BMR is the basal metabolic rate assumed to be 1.2 J/kg equivalent to 3.4 ml O_2_/kg/min using 1 ml O_2_ equivalent to 20.9 J, *A* is the capacity of the anaerobic metabolism in J/kg, *E* is the reduction in peak aerobic power with natural logarithm of race duration *T* (when *T* > *T*_MAP_), MAP is the maximum aerobic power in W/kg, *f* is a constant describing reduction in energy from anaerobic metabolism over time *T*, and *T*_MAP_ is the time for which the MAP can be sustained (assumed to be 420 s).

The Peronnet-Thibault model was able to estimate world record performances ranging from 60 m to full marathons with an average absolute error of 0.73% for males and 1.27% for females. The limitations, however, include the determination of the parameters *A*, MAP, and *E* as well as the accuracy of the assumed parameters such as BMR and *T*_MAP_ in Eq. 8. Morton [51] also discusses a bioenergetic hydraulic model with separate cases for maximal power, endurance at constant work rate, and endurance at incremental ramp exercises comprising of several parameters. Morton compares the bioenergetic model estimates to experimental data from other studies and shows them to be in agreement in predicting the endurance time for the different cases [51]. These alternate models, though having better accuracy in predicting the maximal instantaneous power and the maximal aerobic power compared to the two-parameter model, have many parameters that need to be assumed or estimated, which adds to their complexity [35, 52]. To address the limitations of the two-parameter model and to reduce the complexity of alternate models, Morton [52] proposed an extension of the two-parameter model by adding a non-zero time asymptote *k* to Eq. 5. This model was called the three-parameter model and is given by:

9 $$ t=\frac{W\hbox{'}}{P- CP}+k $$

where k can be derived by substituting *t* = 0 and *P* = *P*_max_ in Eq. 9 resulting in

10 $$ k=\frac{W\hbox{'}}{CP-{P}_{\mathrm{max}}} $$

Rewriting Eq. 9 as

11 $$ t=\frac{W\hbox{'}}{P- CP}+\frac{W\hbox{'}}{CP-{P}_{\mathrm{max}}} $$

results in *k* < 0 as *P*_max_ > CP. The *P*_max_ term in Eq. 11 represents the point at which the power curve intersects the power axis representing an instantaneous maximum power that can be produced.

Weyand and colleagues [53] proposed a model for all-out cycling efforts ranging between 3 and 300 s which is similar to that of Ward-Smith’s [3] given by:

12 $$ P(t)={P}_{\mathrm{aer}}+\left({P}_{\mathrm{mech}\;\max }-{P}_{\mathrm{aer}}\right)\cdot {e}^{\left(-{k}_{\mathrm{cycle}}\cdot t\right)} $$

where *P*_aer_ (synonymous with CP) is the maximum power output that can be supported aerobically, *P*_mech max_ is the maximum power output for a 3-s trial, and *k*_cycle_ is the exponent describing the decrease in power with the increase in time *t*. Morton [54] also provided an extension of the three-parameter model while presenting a model for all-out running efforts given by:

13 $$ P(t)= CP+\left({P}_{\mathrm{max}}- CP\right)\cdot {e}^{\left(t/k\right)} $$

**Fig. 3:**
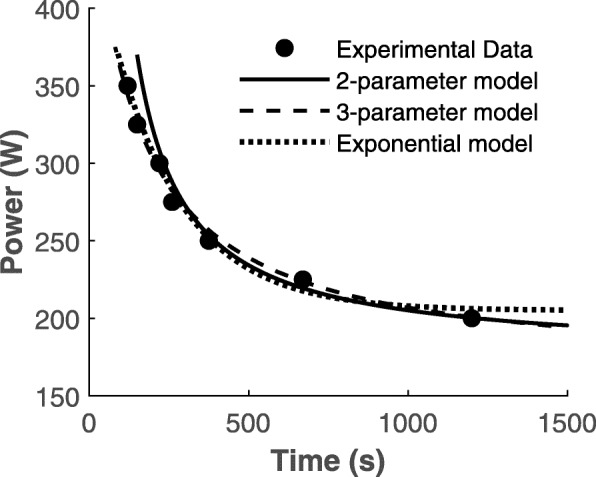
The two-parameter model (solid line), the three-parameter model (dashed line), and the exponential model (dotted line) fitted to the same experimental data (solid circles) presented by Gaesser and colleagues [34]. Data extracted from Fig. 2 in [34] (p. 1434) and redrawn with permission using the values reported in the original article

The model in Eq. 13 is similar to the models proposed by Ward-Smith [3], Hopkins and colleagues [49], and Weyand and colleagues [53]. Expressing Weyand’s *k*_cycle_ as a reciprocal will result in Hopkins’ model in Eq. 7 and Morton’s model in Eq. 13. The signs of these constants are different, which are accounted for by regression. Figure 3 shows three models (two-parameter, three-parameter, and exponential) plotted against experimental data presented by Gaesser and colleagues [34]. The values of CP, *W*′, and *P*_max_ were taken from [34], and data points were extracted using the open source software Plot Digitizer. Table 1 summarizes the estimates from each method.

**Table 1:** Summary of estimates from all models fit to the data presented by Gaesser and colleagues [34]

Model	CP (*W*)	*W*′ (J)	*P*_max_ (W)	Additional model parameters (λ, τ, k_cycle_, or k) (s)
Two-parameter model	1 176	2 29100	3 NA	4 NA
5 Three-parameter model	6 165	7 47900	8 491	9 − 146.93
10 Exponential model	11 205	12 NA	13 452	14 0.0044 or − 225.2867*

*Morton’s [54] *k* = − 225.2867, Hopkins’ [49] *τ* = 225.2867, which are same as Weyand’s [53] − 1/*k*_cycle_ and Ward-Smith’s [3] − 1/*λ*

There are other models proposed in the literature which are algebraic manipulations of the two-parameter model shown in Eq. 5. However, these models yield different estimates of CP and *W*′ at the individual level for the same data as seen in [34, 55, 56]. These differences in estimates could originate from the rounding off approximations of reciprocals such as 1/*t*_Lim_. CP estimates from different models are reported to be in close agreement with each other in [34, 55, 56]. However, as illustrated in Table 1, the estimation of *W*′ remains elusive as the same data can yield different estimates depending on the model used even though CP estimates are comparable [34, 38, 55–61]. The two-parameter model, though having limitations (*P* = ∞ at *t* = 0 and CP lasting indefinitely), owing to its simplicity, can potentially be used to optimize performance as well as determining strategies by estimating time-to-exhaustion [16, 17, 62].

### Methods and Protocols to Estimate CP and *W*′

The first protocol to estimate CP and *W*′ was derived from Monod and Scherrer’s [2] work. Subjects would complete at least three constant work-rate (CWR) to exhaustion tests, and the two-parameter model would then be fit to the data resulting in CP and *W*′ estimates. D. W. Hill [35] suggests the use of the linear model (*P* versus 1/*t*_Lim_) with at least 4–5 CWR tests to arrive at CP and *W*′ estimates.

While less prevalent in the literature, Morton [58] demonstrated another method to determine estimates of CP and *W*′ from ramp exercises to exhaustion by deriving an equation between time-to-exhaustion and ramp slope given by:

14 $$ T=\frac{CP}{S}+\sqrt{\frac{2\cdot W\hbox{'}}{S}} $$

where *T* is the time-to-exhaustion in seconds and *S* is the ramp slope in watts/second. Morton suggested that subjects complete 4–5 ramp tests to exhaustion at different slopes. The time-to-exhaustion from these tests are then plotted against the slopes and Eq. 14 would be fitted to the data to determine CP and *W*′. Morton claims that the estimates from this protocol appear to be lower than those from the CWR protocol thus addressing the overestimation of CP reported in a few publications cited earlier. The ramp protocol was compared to the CWR protocol by Morton and colleagues [63] showing an underestimation of *W*′ and no statistical difference for CP. However, a closer inspection shows underestimation of *W*′ by approximately 10 kJ, 4 kJ, 3 kJ, and 9 kJ for subjects 1, 2, 3, and 6 respectively and an overestimation of *W*′ by approximately 8 kJ, 6 kJ, and 3 kJ for subjects 4, 9, and 10 respectively (see Table 1 in [63]).

**Fig. 4:**
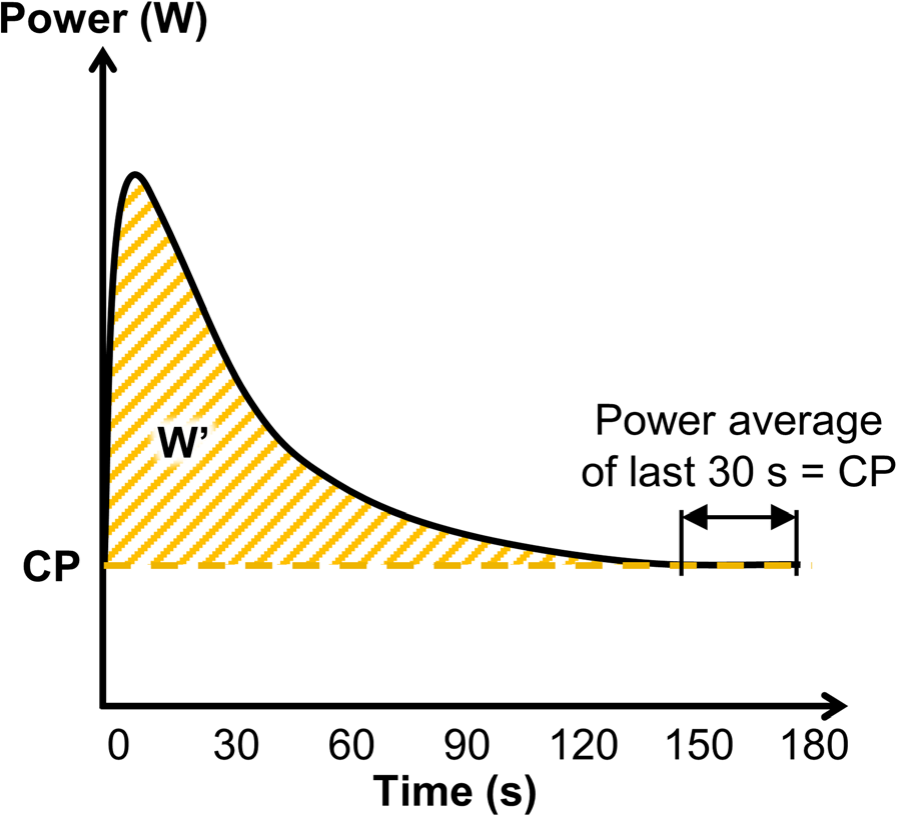
Schematic representation of a 3-min all-out test to determine critical power (CP) and the curvature constant (*W*′). The average power of the last 30s yields CP and the area below the curve and above CP yields *W*′

Vanhatalo and colleagues [64] proposed the 3-min all-out test (3MT) to determine CP and *W*′ in fewer laboratory visits. This test involves pedaling at all-out intensity for 3 min with CP estimated by the average power from the last 30 s and *W*′ given by the area under the curve above CP [57, 64]. Figure 4 shows the schematic representation of a notional 3MT. Parallels can be drawn between the 3MT and the Wingate anaerobic test [65], which is essentially a 30-s all-out test. Studies that compare *W*′ to the anaerobic capacity from the Wingate test report a correlation coefficient of ~ 0.7 [66, 67]. Therefore, the anaerobic capacity from the Wingate test and *W*′ cannot be used interchangeably.

The estimates from the 3MT have been compared to those from the CWR tests in [28, 60, 68] and thereby, validating the 3MT. Burnley and colleagues [69] saw (in 7 out of 11 subjects) a steady state blood lactate and oxygen uptake profile in 30 min of exercise at 15 W below CP determined from the 3MT. They made the same subjects pedal at 15 W above CP which resulted in an average time-to-exhaustion of 13 ± 7 min. Black and colleagues [62] used the CP determined from the 3MT to successfully estimate a 16.1 km time trial performance. However, studies have reported that the time-to-exhaustion at CP derived from the 3MT to be 14.79 ± 8.38 min and 12.5 ± 6.5 min in [70, 71] respectively. These are similar to 13 ± 7 min for exercise at 15 W above CP reported by Burnley and colleagues [69]. Moreover, *W*′ from 3MT has also been reported to be overestimated in comparison to CWR protocol (11.37 ± 3.84 kJ vs 9.55 ± 4 kJ) [72]. However, as discussed by Skiba [73], the errors observed in the estimates could be attributed to not using the same equipment or not adhering to the test procedure laid out in [64]. Additionally, the inherent day-to-day variability within subjects, referred to as the intra-individual variability (IIV), may have contributed to the shorter time-to-exhaustion observed at CP [17, 38]. Hence, exercise outside a subject’s 95% confidence interval of CP, i.e., outside the bounds of the IIV associated with CP (similar to 15 W above and below CP in [69]), will yield better insights into reliability of the 3MT.

### Limitations of the Protocols Used to Determine CP and *W*′

The CWR protocol is considered as the “gold-standard” to estimate CP and *W*′ as it was the first method to be proposed. However, the CWR protocol is not devoid of shortcomings. Using the CWR protocol, Bishop and colleagues [74] and Jenkins and colleagues [75] illustrated that the duration of the predicting trials influences the estimates with both CP and *W*′ computed from three shortest duration trials being significantly greater than those from the three longest trials. Furthermore, CP estimates from the CWR protocol at 60 rpm have been found to be significantly greater than those at 100 rpm [76]. Considering these limitations, Muniz-Pumares and colleagues [61] suggest the use of the two-parameter hyperbolic model with at least three CWR trials of durations > 2 min and < 15 min and freely chosen cadence to arrive at reliable estimates.

The 3MT avoids the need to do multiple tests to arrive at CP and *W*′. However, there are reports of overestimation of CP from the 3MT [70, 71, 77], which are comparable to other reports of overestimation of CP from the CWR tests in [41–44]. The 3MT appears to reliably predict a 16.1 km time trial performance [62], which is in accordance with other studies that report the validity of CP to be 40 min to over 1 h [35, 45, 46]. These contradictory results can be attributed to equipment, test method, validation methods, and the day to day variability of the participants [17, 38, 73].

**Fig. 5:**
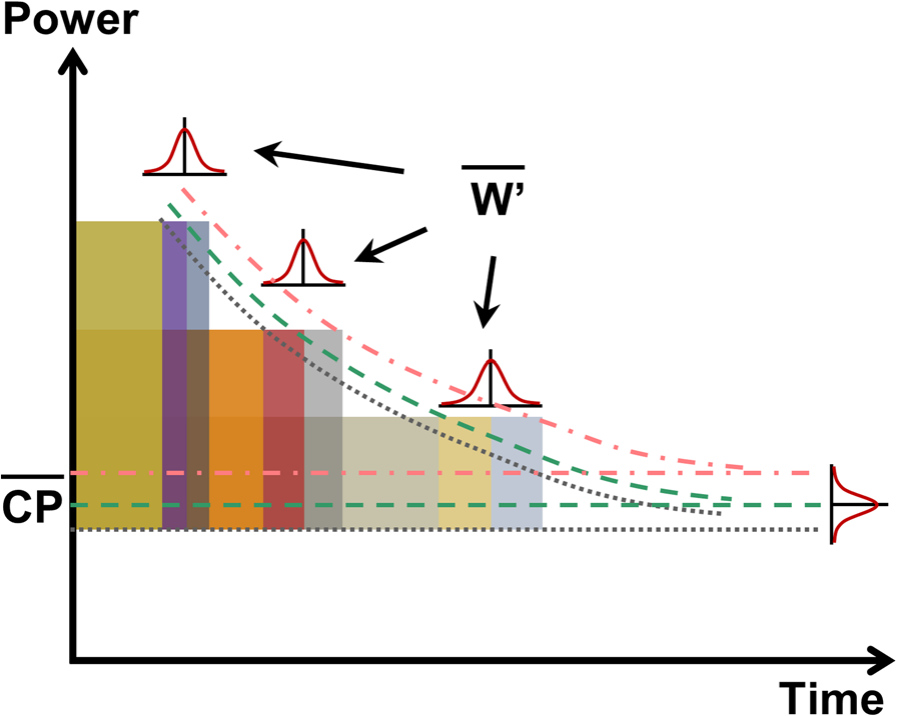
Repeated constant work-rate (CWR) tests to capture intra-individual variability (IIV) associated with critical power (CP) and curvature constant (*W*′) estimates. The dotted, dashed, and dot-dashed lines show the fits to the different sets of data and their respective asymptotes. The grand means for CP and *W*′ are obtained by averaging the respective parameters estimates from each curve fitting

It has been shown that the day-to-day (or trial-to-trial) variability within a person, i.e., IIV, affects performance during physical activities in [78]. The CWR tests, depending on the fit and the model used, yield standard errors of estimation (SEEs) for CP and *W*′. These SEEs give a measure of goodness of fit and not the IIV. To truly capture and quantify IIV using the CWR protocol, exercise to exhaustion at each work-rate must be repeated multiple times. CP and *W*′ estimates for each set of tests could be determined, which can then be averaged to arrive at a grand mean for CP and *W*′ (see Fig. 5). On similar lines, Triska and colleagues [79] conducted maximal effort time trials spanning 3, 7, and 12 min with each trial repeated thrice (one familiarization and two repeats) and computed CP and *W*′ for each data set using the two-parameter hyperbolic model. They found higher reliability between the post familiarization trials with intra-class correlation coefficient of 0.95 and 0.94 and a coefficient of variation of 2.6% and 8.2% for CP and *W*′ respectively. However, an average CP and *W*′ for all three sets of data (or post familiarization trials) could be computed to yield grand means for CP and *W*′ for each subject with their IIVs as shown in Fig. 5. Although costly in terms of time, this method may lead to a better understanding of *W*′, which has been shown to be ambiguous and significantly dependent on the mathematical model used [34, 55–57, 59–61].

**Fig. 6:**
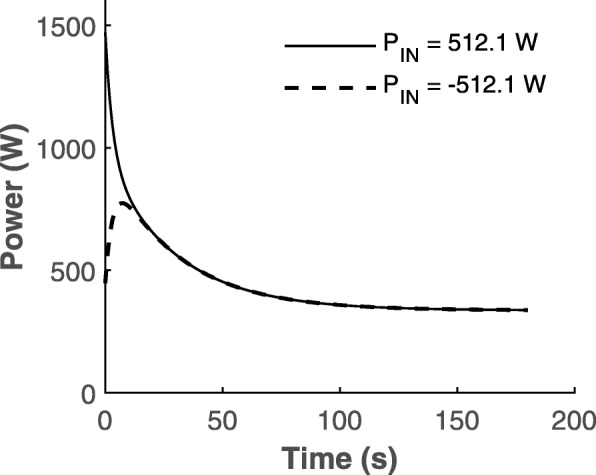
Morton’s biexponential model [54] plots showing positive inertial resistance of ergometer flywheel, *P*_IN_ (solid line) and negative *P*_IN_ (dashed line). The positive *P*_IN_ term does not yield the shape shown in Fig. 4

Though the 3MT has been shown to be repeatable in [69], a closer investigation of the Bland-Altman plots presented in the first paper on 3MT [69] (p.1998, Fig. 1d) shows the bias and 95% limits of agreement of − 1 ± 15 W resulting from the variability associated with each subject’s CP estimate across two trials. A 15-W change in CP between two 3MTs contributes to a difference of 2700 J of *W*′ across the 3 min of the test. This IIV needs to be accounted for before prescribing training schedules and interventions based on the 3MT. The estimates from the CWR protocol have associated SEEs for CP and *W*′, whereas it is not possible to get a standard error for *W*′ from a 3MT. A possible way to arrive at SEEs for CP and *W*′ from the 3MT is by fitting a curve to the data. Morton [54] used a biexponential extension to his exponential model (Eq. 13) to be applicable to all-out efforts given by,

15 $$ P(t)= CP+\left({P}_{\mathrm{max}}- CP\right)\cdot {e}^{t/k}+{P}_{\mathrm{IN}}\cdot {e}^{-t/k\hbox{'}} $$

where *P* is the power at any time *t*, CP is the critical power, *P*_max_ is the instantaneous maximum power, *P*_IN_ is the power required to overcome the initial inertial resistance of the ergometer flywheel, and *k* and *k*′ are constants. The *P*_IN_ term accounts for 0–-5 s of the all-out test. The model in Eq. 15 is shown to fit the all-out test data with *R*^2^ = 0.985 in [54]. However, it has the following shortcomings:

- At *t* = 0, *P*(0) = *P*_max_ + *P*_IN_, which is not possible as the instantaneous maximum power that can be generated is *P*_max_. Instead, at *t* = 0, *P*(0) = *P*_max_ − *P*_IN_ is a more realistic power output. The *P*_max_ – *P*_IN_ correction is a mathematical quirk and lacks physiological basis. However, *P*_max_ could be assumed to be equal to either the average power output of one crank-rotation [40] or the power output of 3-s trial [53] which accounts for the physiological constraints of producing an instantaneous *P*_max_. Furthermore, if the all-out interval starts from rest, then at *t* = 0, *P*(0) = 0 is a more valid initial condition as power is defined as energy-expended/time and no energy is expended before starting the exercise.
- Morton fit the model to Burnley’s data in [69] which resulted in the CP = 336.3 ± 1.2 W, *P*_max_ = 959.3 ± 7.9 W, *P*_IN_ = 512.1 ± 13.8 W, *k* = − 29.9 ± 0.5 s, and *k*′ = 3.14 ± 0.16 s. Using these values in Eq. 15 and plotting against time (from 0 to 180 s) does not result in the desired shape of the 3MT as shown in Fig. 4 (see Fig. 6). If *P*_IN_ were to be negative, the resulting shape would be similar to that of Fig. 4. However, a negative resistance for the flywheel is unrealistic.
- The power required to overcome the inertial resistance of the flywheel can be computed using Newton’s second law for rotational motion as shown in [80]. The *P*_IN_ term is a function of torque and acceleration. Thus, there is no reason to assume an exponential decay as shown in Eq. 15. A piecewise model could be developed for a 3MT with the first piece to account for the power needed to overcome the flywheel’s inertia and the second to account for the decline from peak power to CP. Furthermore, the time taken by the muscle to reach *P*_max_ needs to be accounted for in the first piece where the muscles are overcoming the flywheel resistance while reaching their maximal power output.

The SEEs from curve fitting, as mentioned earlier, do not quantify the IIV associated with CP and *W*′ for an individual. Conducting multiple tests and computing grand means for CP and *W*′ from each set of tests significantly increases the time investment. There is a need for better methods to capture the IIV from a 3MT, minimize the number of testing days, and statistically compare two 3MTs to arrive at reliable estimates of CP and *W*′ for an individual. Furthermore, most studies report the average of their participant groups. While this is convenient in terms of comparing them with estimates from other methods and protocols, they give little information pertaining to the repeatability and variability at the individual level. It is, therefore, practical to consider individuals rather than groups and arrive at athlete-specific models. This is important in terms of modeling recovery of *W*′ which could be appended to the two-parameter CP model, thereby aiding in performance optimization.

### Adding Recovery to the Two-Parameter Model

**Fig. 7:**
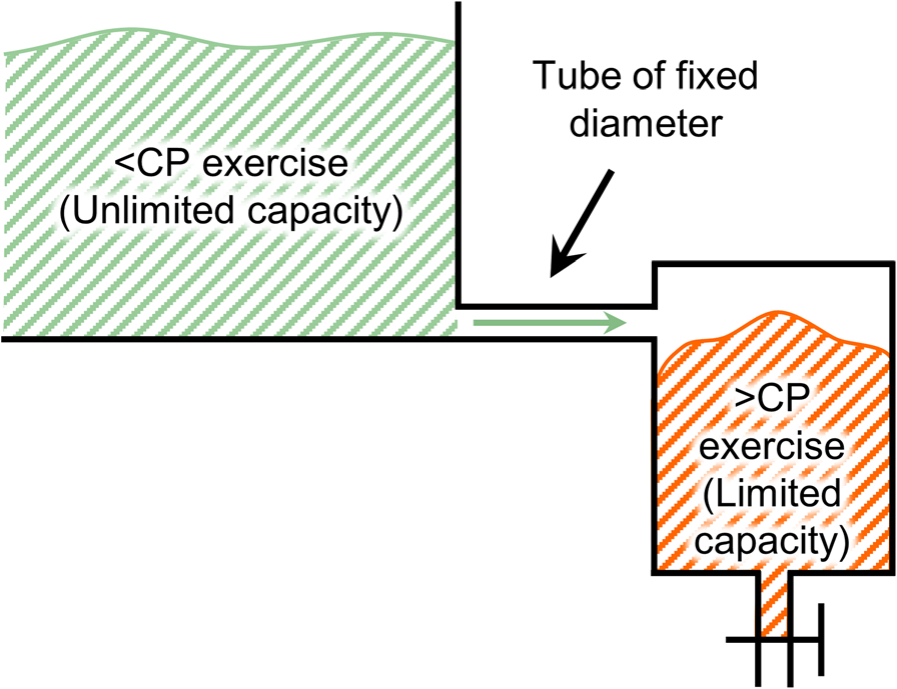
Critical power (CP) concept using Morton’s hydraulic vessel analogy [38]: Energy domains show sub-CP and supra-CP vessels connected by a tube of fixed diameter. Morton’s aerobic and anaerobic vessels are replaced by < CP and > CP respectively as the curvature constant (*W*′) and anaerobic work capacity (AWC) cannot be used interchangeably

The CP concept has been discussed using a hydraulic vessel analogy by Morton [38]. Morton [38] discusses that the aerobic and anaerobic domains are analogous to energy vessels connected by a tube of fixed diameter, with the anaerobic vessel being limited in capacity and the aerobic being unlimited (see Fig. 7). Morton suggests that when functioning above CP, energy is derived from the anaerobic vessel, whereas when exercising below CP, energy is supplied by the aerobic vessel. Morton’s hydraulic analogy considers CP to be the boundary between aerobic and anaerobic domains, and AWC to be equal to *W*′ as it was published around the same time as Dekerle and colleagues’ study [33] that showed that AWC and *W*′ cannot be used interchangeably.

Ignoring the assumption of AWC and *W*′ being equal, Morton’s analogy suggests that while below CP, the curvature constant *W*′ (limited capacity tank in Fig. 7) is refilled or recovered. This suggestion presents the possibility of modeling the recovery of *W*′ while exercising below CP and thereby, together with the two-parameter model, optimizing performance. While there are models to estimate the depletion of *W*′, there are only a few models that attempt to estimate its recharge/recovery while below CP.

The first model considering recovery of *W*′ was proposed by Morton and Billat [81]. Morton and Billat [81], based on the two-parameter model, derived an equation for time-to-exhaustion for intermittent exercise by assuming that the rates of recharge and expenditure of *W*′ were equal given by:

16 $$ t=\frac{n\cdot \left({t}_w+{t}_r\right)+W\hbox{'}-n\cdot \left[\left({P}_w- CP\right)\cdot {t}_w-\left( CP-{P}_r\right)\cdot {t}_r\right]}{\left({P}_w- CP\right)} $$

where *t* is the total duration of the intermittent exercise, *n* is the number of intervals, *t*_w_ and *t*_r_ are respective durations of intervals above and below CP, and *P*_w_ and *P*_r_ are respective power outputs of intervals above and below CP. Ferguson and colleagues [82] were first to quantify recovery of *W*′ by proposing that it is “curvilinear” and not proportional to its depletion as assumed by Morton and Billat [81]. Acknowledging this curvilinear nature of recovery of *W*′, Skiba and colleagues [83–86] proposed a model which assumed the behavior to be exponential given by:

17 $$ W{\hbox{'}}_{bal}=W\hbox{'}-\underset{0}{\overset{t}{\int }}W{\hbox{'}}_{\mathrm{exp}}\cdot {e}^{-\left(\frac{\left(t-u\right)}{\tau_{W\hbox{'}}}\right)} du $$

where *W*′_bal_ is the *W*′ balance at any time during exercise, *W*′_exp_ is the amount of *W*′ expended, (*t* − u) is the duration of the recovery interval, and *τ*_*W*′_ is the time constant of reconstitution of *W*′ in seconds given by:

18 $$ {\tau}_{W\hbox{'}}=546\cdot {e}^{\left(-0.01{D}_{CP}\right)}+316 $$

where *D*_CP_ is the difference between CP and average power output during all intervals below CP. Eq. 18 is a non-linear regression obtained by plotting *τ*_*W*′_ values (calculated by setting the *W*′_bal_ = 0 in Eq. 17 at the termination of exercise) against respective *D*_CPs_.

Skiba’s model was validated in [84] where an average *W*′ balance at exhaustion of 0.5 ± 1.3 kJ was reported. However, the model cannot be used to determine *W*′ balance in real time [73] (p.78) as the τ_*W*′_ term needs *W*′_bal_ to be zero which is not known until the termination of each test. Moreover, three forms of the *W*′_bal_ model have been published by Skiba and colleagues [83–86]. The first [83] contains only the integrand and not the differential variable. The second [84, 85] contains the differential “du” as shown in Eq. 17, whereas the third [86] has “dt” as its differential variable. Changing the differential variable from “du” to “dt” yields different results upon integration. Additionally, inspecting Eq. 17 reveals that the integral term on the right-hand side has units of Joules-second causing an inequality as the units of the left-hand side are Joules. Additional file 1 of this manuscript provides a detailed derivation of the mathematical solutions for both “du” and “dt” as the differential term of the *W*′_bal_ model illustrating the difference in results as well as the imbalance of units. Furthermore, the standard errors associated with the estimation of CP and *W*′ may cause a negative balance of *W*′ balance (can be seen in [84], Fig. 2, p.903). Skiba and colleagues [85] proposed a biconditional *W*′_bal_ model which resolves the inequality of units (can be seen in Appendix 1 of [85]) given by:

19 $$ {\displaystyle \begin{array}{l}\mathrm{If}\;P> CP,\kern1em W{\hbox{'}}_{\mathrm{bal}}=W{\hbox{'}}_0-\left[\left(P- CP\right)\cdot t\right]\\ {}\mathrm{If}\;P< CP,\kern1em W{\hbox{'}}_{\mathrm{bal}}=W{\hbox{'}}_0-W{\hbox{'}}_{\mathrm{exp}}\cdot {e}^{\left(\frac{-{D}_{CP}\cdot t}{W{\hbox{'}}_0}\right)}\end{array}} $$

where *W*′_0_ is *W*′ at time *t* = 0. Though the model in Eq. 19 resolves the inequality of units, it has been shown to underestimate the recovery of *W*′ by Bartram and colleagues [87]. Bickford and colleagues [88] presented a model of recovery of *W*′ which was derived from limited data and thus needs refinement.

Apart from the models presented above, at the time of submission, there are no models available in the literature that attempt to model the recovery of *W*′. These models need to be improved for accurately modeling the recovery of *W*′ and combining them with the models of exertion that are well established in the literature. There is potential in extending the two-parameter model to include the recovery model. A combined exertion-recovery/discharge-recharge model of *W*′ will be worthwhile in estimating the time-to-exhaustion of endurance efforts and optimizing performance. The potential of optimizing performance to accomplish a 2-h marathon has been illustrated by Nike’s Breaking2 project [89] which has inspired modeling studies by Hoogkamer and colleagues [90–92] based on the two-parameter CP model with exponential recovery similar to Eq. 19, biomechanical improvements, shoe design improvements, and drafting strategy. Furthermore, the successful completion of a sub 2-h marathon by Eliud Kipchoge as a part of the INEOS 1:59 challenge in Vienna in October 2019 provides encouraging signs for investigative studies focusing on optimization of performance in other endurance sports.

#### Applications of a Combined Expenditure-Recovery Model of *W*′

In the literature reviewed thus far, studies modeling recovery of *W*′ are scarce. A few models attempt to address the need for a combined expenditure-recovery model. Skiba’s first model [83] is similar to the mono-exponential ventilatory gas exchange model for moderate intensity cycling proposed by Whipp and colleagues [93] and Vandewalle and colleagues’ aerobic power model [67]. The exponential assumption of recovery seems logical as sub-CP exercise is considered to be supported by aerobic mechanisms [38]. The *τ*_*W*′_ relation in Eq. 18 is representative of the seven recreational athletes from whose data it was derived. Though the model was validated using data from eight triathletes [84], it may not be able to predict the recovery of *W*′ for athletes of higher or lower caliber. This is illustrated by Caen and colleagues [94] where faster recovery of *W*′ was observed. Skiba’s second model (also mono-exponential) [85], derived from first principles with valid assumptions, addresses some limitations of the earlier version. However, it has not been validated and, like its predecessor, has been shown to have slower recovery kinetics for elite athletes by Bartram and colleagues [87].

De Jong and colleagues [95] have used the two-parameter model to simulate the optimization of a 5-km time-trial performance. However, a recovery model in combination with the two-parameter model will aid in optimizing performance over longer durations and distances. There have been other attempts at combining the two-parameter model with a recovery model [88], but the limited data result in the need for refinement. The advantage of an exertion-recovery model is the ability to accurately predict the time-to-exhaustion during endurance exercises. Furthermore, modeling fatigue, exhaustion, and recovery has applications not only in the field of athletic training and performance but also in the fields of medicine and health monitoring [12, 16, 17].

With an exertion-recovery model based on the CP concept, an energy management system can be designed that will regulate the expenditure and recovery of *W*′. The optimization objectives would be minimizing time and maximizing distance by maximizing power output with the help of an exertion-recovery model. For example, in cycling races, 3–4 cyclists form pelotons to reduce drag. It has been shown that the cyclists in the middle of a peloton experience up to 40% less drag [96]. A potentially successful race strategy for the peloton group can be derived from the exertion-recovery model using CPs and *W*′s of the individual riders. A similar drafting strategy was employed by Eliud Kipchoge in the INEOS 1:59 challenge where he completed a full marathon in 1 h 59 min and 40.2 s. Another application is an energy management system for foot missions of soldiers. Time to exhaustion in long foot missions, where soldiers carry all the load of ammunition, food, and water can be accurately estimated with an exertion-recovery model. Additionally, in team sports like football, rowing, lacrosse, and soccer, CP and *W*′ could be used in team selection, determining team strategies, planning individual training needs, and training interventions [97]. Furthermore, the combined model can be used to link *W*′ balance to performance quality and to estimate injury risk. Together with wearable sensors, the model could potentially be used to determine team strategies in terms of player substitutions and avoiding fatigue-related injuries and for real-time performance optimization. The rise in popularity of wearable sensors has resulted in their use in health monitoring [98] and physical activity tracking [98, 99] and provides opportunities to mitigate dependence on laboratory equipment. Therefore, models of human performance can be tested and validated outside the laboratory.

### Research Opportunities in Modeling Human Performance

The research opportunities identified in this review article are cross-functional encompassing the areas of human performance, exercise physiology, health, and engineering. Though the themes belong to different backgrounds, they are not independent of each other. Table 2 summarizes the theme-wise research opportunities and applications that have been identified in this paper.

**Table 2:** Theme-wise research opportunities and applications of human performance modeling

Themes	Research opportunities and applications
Groups versus individuals	Models derived from the data pertaining to a group of individuals may not accurately model performance of athletes outside the group, thus, suggesting a need for individual specific models [87].
Influence of mathematical modeling on *W*′	Understanding of *W*′ is still ambiguous as it is dependent on the model used [34, 55–57, 59–61]. Quantifying the natural day-to-day/trial-to-trial variability within subjects, i.e., IIV, may yield a better understanding of *W*′.
Natural variability within an individual	Methods need to be developed to quantify the IIV associated with physiological parameters, which will be useful in measuring training effectiveness, developing higher fidelity models, and optimizing performance.
Recovery of *W*′	Current models described in [83–88] need refinement and improvement. A robust model for recovery of *W*′ is needed, which could be athlete-specific. The *W*′ balance can potentially be correlated to fatigue related injuries and the risk of injury could be estimated.
Performance optimization	The recovery model in conjunction with the two-parameter model enables optimization of time-trial performance as illustrated in [95, 100] and illustrated in [91, 92].
Wearable sensor integration	Wearable sensors provide opportunities in real-time performance tracking, optimization, and methods to reduce the reliance on laboratory equipment. Similar to studies in [101, 102], commercially available sensors could be validated against laboratory equipment and used in the field for developing higher fidelity models.
Integration of individual performance modeling into team performance	Athlete-specific models could be used in determining team strategies, training interventions, planning training needs, and team selection as illustrated in [91, 97].
Physical exertion and health	Models of human performance could be used to gain insight into the effect of physical exertion on overall health and well-being as discussed in [16, 17].

Developing mathematical models of fatigue will not only aid athletes, but also defense personnel in mission planning and healthcare professionals who study the effect of physical exertion on overall health. The ability to quantify the day-to-day variability aids the measurement of training effectiveness and training prescription. Furthermore, the theory of expenditure of *W*′ is explained well by the two-parameter model. However, a robust model for recovery of *W*′ is yet to be proposed.

## Conclusions

The objective of this paper was to review the state of the art for power-based models of fatigue and identify opportunities to advance the field. Power based models of human performance which have their origins in cycling have been reviewed. The two-parameter CP concept reliably estimates fatigue due to severe intensity exercise in the range of 2 min to 1 h and is also suitable to model sprint performances of appropriate durations. Alternate models predict the power and time relationship in the severe intensity domain with better accuracy, but these models require the determination of more parameters, thereby, increasing complexity. CP and *W*′ can be estimated using multiple models and protocols with the 3MT being the least time-consuming method. The 3MT, despite its advantages, has a limitation of not capturing the IIV associated with CP and *W*′ estimates. Standard errors associated with the estimates from the power-time regression of CWR tests could help in better quantifying this variability. However, they only give a measure of goodness of fit and do not capture the IIV. None of the models available accommodate the IIV associated with the parameter estimates, regardless of the method of estimation used. Until methods to capture IIV are proposed and validated, subject-specific training prescription and subsequent performance optimization will be limited in precision and accuracy. Additionally, models derived from group data do not represent the population as several factors and variables have a bearing on human performance. Individualized athlete-specific models need to be derived to potentially improve performance through training prescriptions. The CP concept, owing to its simplicity, is promising and robust in terms of modeling fatigue in the severe intensity domain. However, it is incomplete due to the lack of proper understanding of the recovery behavior of *W*′ in the moderate and heavy intensity domains. Attempts have been made to address this gap, but with limited success. The models available provide a good starting point to develop models of higher accuracy and fewer assumed parameters. A combined exertion-recovery model will lead to optimized performance realized through an energy management control system. The combined model could lead to a straightforward way of assessing fatigue and risk of injury and have implications with respect to the influence of exercise on overall health.

## Supplementary information

**Additional file 1. **Derivation of mathematical solutions for the different forms of the *W*′_bal_ model presented by Skiba and colleagues.

## Data Availability

Not applicable as data were neither generated nor analyzed in the current study. The supplementary material contains detailed mathematical solutions of the different forms of the *W*′_bal_ model.

## Abbreviations

3MT: 3-min all out test
AWC: Anaerobic work capacity
BMR: Basal metabolic rate
CP: Critical power
CV: Critical velocity
CWR: Constant work-rate
*D*′: Curvature constant of velocity-time relationship
*D*_CP_: Difference between critical power and the average recovery power
GET: Gas exchange threshold
IIV: Intra-individual variability
LT: Lactate threshold
MAP: Maximum aerobic power
MLSS: Maximal lactate steady state
*P*_aer_: Maximum power output supported aerobically
*P*_IN_: Power to overcome the inertia of the flywheel of the ergometer
*P*_max_: Instantaneous maximum power;
*P*_mech max_: Maximum power output for a 3 s trial
SEE: Standard error of estimation
*t*_Lim_: Time to exhaustion
*T*_MAP_: Time to exhaustion at maximum aerobic power
*V*_max_: Maximum muscle shortening velocity
V̇O_2_: Oxygen uptake
V̇O_2max_: Maximal oxygen uptake
*W*′: Curvature constant of the power-time relationship
*W*′_bal_: *W*′ balance
*W*_Lim_: Limit work
