# Supplementary material for: A survey of mathematical models of human performance using power and energy
Source: Sports Med Open. 2019 Dec 27;5:54. doi: 10.1186/s40798-019-0230-z (PMC6934642; doi:10.1186/s40798-019-0230-z)
Supplement: Supplementary file 1 — Additional file 1. Derivation of mathematical solutions for the different forms of the W′bal model presented by Skiba and colleagues. [file 40798_2019_230_MOESM1_ESM.docx]

**Supplementary material for the paper titled: A survey of mathematical models of human performance using power and energy.**

Authors: Vijay Sarthy M Sreedhara^1^, Gregory M Mocko^1^, and Randolph E Hutchison^2^

^1^Department of Mechanical Engineering, Clemson University, Clemson, SC 29634, USA

^2^Department of Health Sciences, Furman University, Greenville, SC 29613, USA

This supplementary material shows the derivation of mathematical solutions for the different forms of the W’_bal_ model presented by Skiba and colleagues [1–4]

W’_bal_ model with only the integrand [1]:

| $W'_{bal}=W'-\int_{0}^{t} W'_{\exp}\cdot e^{-\left(\frac{(t-u)}{\tau_{W'}}\right)}$ |  |
| --- | --- |

W’_bal_ model with *du* as the differential variable [2,3]:

| $W'_{bal}=W'-\int_{0}^{t} W'_{\exp}\cdot e^{-\left(\frac{(t-u)}{\tau_{W'}}\right)}du$ |  |
| --- | --- |

W’_bal_ model with *dt* as the differential variable [4]:

| $W'_{bal}=W'-\int_{0}^{t} W'_{\exp}\cdot e^{-\left(\frac{(t-u)}{\tau_{W'}}\right)}dt$ |  |
| --- | --- |

In all the forms, W’_bal_ is the W’ balance at any time during exercise, W’_exp_ is the amount of W’ expended, (t − u) is the duration of the recovery interval, and τ_W’_ is the time constant of reconstitution of W’ in seconds given by,

| $\tau_{W'}=546\cdot e^{(-0.01D_{CP})}+316$ |  |
| --- | --- |

where, D_CP_ is the difference between CP and average power output during all intervals below CP (recovery power). Equation 2 is a non-linear regression obtained by plotting τ_W’_ values (calculated by setting the W’_bal_=0 in Equation 1 at the termination of exercise) against respective D_CPs_.

Equation 1 cannot be integrated due to the absence of the differential term. Hence, in the following section Equations 2 and 3 will be integrated to show the difference in the obtained solution. Additionally, dimensional analyses will be conducted for both solutions to show the imbalance of units.

## Integration of Equations 2 and 3

In both the following integrations, the W’_exp_ term is treated as a constant.

### Integration of version 2

Rewriting Equation 2,

$$W'_{bal}=W'-\int_{0}^{t} W'_{\exp}\cdot e^{-\left(\frac{(t-u)}{\tau_{W'}}\right)}du$$

Treating W’_exp_ as a constant,

$$W'_{bal}=W'-W'_{\exp}\cdot\int_{0}^{t} e^{-\left(\frac{(t-u)}{\tau_{W'}}\right)}du$$

Integrating the exponential term with respect to u,

$$\begin{matrix} W'_{bal}=W'-\left[W'_{\exp}\cdot\left\{\tau_{W'}\left(e^{\frac{u-t}{\tau_{W'}}}\right)_{u=0}^{u=t}\right\}\right] \\ =W'-W'_{\exp}\cdot\tau_{W'}\left(e^{\frac{t-t}{\tau_{W'}}}-e^{\frac{0-t}{\tau_{W'}}}\right) \\ W'_{bal}=W'-W'_{\exp}\cdot\tau_{W'}\left(1-e^{\frac{-t}{\tau_{W'}}}\right) \end{matrix}$$

#### Dimensional analysis of the integration of version 2

The result of the integration of Equation 2 is

| $W'_{bal}=W'-W'_{\exp}\cdot\tau_{W'}\left(1-e^{\frac{-t}{\tau_{W'}}}\right)$ |  |
| --- | --- |

Units of the terms on the left-hand-side (LHS): W’_bal_ is in Joules (J)

Units of the terms on the right-hand-side (RHS): W’ is in J, W’_exp_ is in J, τ_W’_ is in seconds (s). The exponential term is dimensionless as the unit of measurement for both t and τ_W’_ is seconds.

Therefore, the units result as:

| $\text{J}=\text{J}-\text{J}\cdot\text{s}\cdot\left(1-e^{\frac{\text{s}}{\text{s}}}\right)$ $\text{J}=\text{J} - \text{J}\cdot\text{s}$   |  |
| --- | --- |

The RHS cannot be computed due to the imbalance of the units.

### Integration of version 3

Rewrtiting Equation 3,

$$W'_{bal}=W'-\int_{0}^{t} W'_{\exp}\cdot e^{-\left(\frac{(t-u)}{\tau_{W'}}\right)}dt$$

Treating W’_exp_ as a constant,

$$W'_{bal}=W'-W'_{\exp}\cdot\int_{0}^{t} e^{-\left(\frac{(t-u)}{\tau_{W'}}\right)}dt$$

Integrating the exponential term with respect to t,

$$\begin{matrix} W'_{bal}=W'-\left[W'_{\exp}\cdot\left\{\tau_{W'}\left(-e^{\frac{u-t}{\tau_{W'}}}\right)_{t=0}^{t=t}\right\}\right] \\ =W'-W'_{\exp}\cdot\tau_{W'}\cdot\left(-e^{\frac{u-t}{\tau_{W'}}}+e^{\frac{u-0}{\tau_{W'}}}\right) \\ =W'-W'_{\exp}\cdot\tau_{W'}\cdot\left(-e^{\frac{u-t}{\tau_{W'}}}+e^{\frac{u}{\tau_{W'}}}\right) \\ W'_{bal}=W'-W'_{\exp}\cdot\tau_{W'}\cdot e^{\frac{u}{\tau_{W'}}}\left(1-e^{\frac{-t}{\tau_{W'}}}\right) \end{matrix}$$

#### Dimensional analysis of the solution to version 3

The result of the integration of Equation 3 is

| $W'_{bal}=W'-W'_{\exp}\cdot\tau_{W'}\cdot e^{\frac{u}{\tau_{W'}}}\left(1-e^{\frac{-t}{\tau_{W'}}}\right)$ |  |
| --- | --- |

Units of the terms on LHS: W’_bal_ is in J

Units of the terms RHS: W’ is in J, W’_exp_ is in J, τ_W’_ is in s. The exponential term is dimensionless as the unit of measurement for u, t, and τ_W’_ is seconds.

Therefore, the units result as:

| $\text{J}=\text{J}-\text{J}\cdot\text{s}\cdot e^{\frac{\text{s}}{\text{s}}}\cdot\left(1-e^{\frac{\text{s}}{\text{s}}}\right)$ $\text{J}=\text{J} - \text{J}\cdot\text{s}$   |  |
| --- | --- |

The RHS cannot be computed due to the imbalance of the units, which is the same as the results of the dimensional analysis of version 2 in Equation 6.

# References

1. Skiba PF, Chidnok W, Vanhatalo A, Jones AM. Modeling the Expenditure and Reconstitution of Work Capacity above Critical Power. Med Sci Sports Exerc. 2012;44:1526–32.

2. Skiba PF, Clarke D, Vanhatalo A, Jones AM. Validation of a novel intermittent W’ model for cycling using field data. Int J Sports Physiol Perform. 2014;9:900–4.

3. Skiba PF, Fulford J, Clarke DC, Vanhatalo A, Jones AM. Intramuscular determinants of the ability to recover work capacity above critical power. Eur J Appl Physiol. 2015;115:703–13.

4. Skiba PF, Jackman S, Clarke D, Vanhatalo A, Jones AM. Effect of work and recovery durations on W’ reconstitution during intermittent exercise. Med Sci Sports Exerc. 2014;46:1433–40.
